# Chemical Composition and Biological Activities of Trans-Himalayan Alga *Spirogyra porticalis* (Muell.) Cleve

**DOI:** 10.1371/journal.pone.0118255

**Published:** 2015-02-18

**Authors:** Jatinder Kumar, Priyanka Dhar, Amol B. Tayade, Damodar Gupta, Om P. Chaurasia, Dalip K. Upreti, Kiran Toppo, Rajesh Arora, M. R. Suseela, Ravi B. Srivastava

**Affiliations:** 1 Defence Institute of High Altitude Research, Defence Research & Development Organisation, Leh-Ladakh, Jammu & Kashmir, India; 2 Medicinal and Aromatic Plants Laboratory, Radiation Biotechnology Group, Institute of Nuclear Medicine and Allied Sciences, Defence Research and Development Organisation, Brig S. K. Mazumdar Marg, Delhi, India; 3 Office of the Director General-Life Sciences, DRDO Bhawan, Rajaji Marg, New Delhi, India; 4 National Botanical Research Institute, Rana Pratap Marg, Lucknow, Uttar Pradesh, India; University of Sassari, ITALY

## Abstract

The freshwater alga *Spirogyra porticalis* (Muell.) Cleve, a filamentous charophyte, collected from the Indian trans-Himalayan cold desert, was identified on the basis of morpho-anatomical characters. Extracts of this alga were made using solvents of varying polarity *viz.* n-hexane, acetonitrile, methanol and water. The antioxidant capacities and phenolic profile of the extracts were estimated. The methanol extract showing highest antioxidant capacity and rich phenolic attributes was further investigated and phytochemical profiling was conducted by gas chromatography-mass spectrometry (GC/MS) hyphenated technique. The cytotoxic activity of methanol extract was evaluated on human hepatocellular carcinoma HepG2 and colon carcinoma RKO cell lines. The anti-hypoxic effect of methanol extract of the alga was tested on *in vivo* animal system to confirm its potential to ameliorate oxidative stress. The antioxidant assays *viz.* ferric reducing antioxidant power (FRAP), 2,2'-azinobis-(3-ethylbenzothiazoline-6-sulfonic acid) diammonium salt (ABTS), 1,1-diphenyl-2-picrylhydrazyl (DPPH) and nitric oxide (NO) radical scavenging capacities, β-carotene-linoleic acid bleaching property and lipid peroxidation exhibited analogous results, wherein the algal extracts showed significantly high antioxidant potential. The extracts were also found to possess high content of total proanthocyanidin, flavonoid and polyphenol. GC/MS analysis revealed the presence of thirteen chemotypes in the methanol extract representing different phytochemical groups like fatty acid esters, sterols, unsaturated alcohols, alkynes etc. with substantial phyto-pharmaceutical importance. The methanol extract was observed to possess anticancer activity as revealed from studies on HepG2 and RKO cell lines. In the present study, *S. porticalis* methanol extract also provided protection from hypoxia-induced oxidative stress and accelerated the onset of adaptative changes in rats during exposure to hypobaric hypoxia. The bioactive phytochemicals present in this trans-Himalayan alga are of enormous interest and can be utilized sustainably for discovery of novel drugs against oxidative stress.

## Introduction

Natural antioxidants present in plants are known to play a vital role against stress-induced oxidative damage resulting due to the production of reactive oxygen species (ROS) within the cellular milieu, or due to various diseases and ageing processes [1–4]. Consequently, such compounds reflect potential in promoting overall health and well-being, and hold potential for medicinal use, particularly for treating a broad range of ailments and maladies confronting humans. As a consequence, various natural plant forms like micro- and macro-algae, lichens, macromycetes and plants etc. have become the major bioresources for natural antioxidants which find substantial use for preventing and treatment of a number of diseases. Algae are of our prime interest in the search for natural antioxidants as they have been reported to possess significant antioxidant activity and free radical scavenging properties [5,6].

Algae are a genetically diverse group of plants that possess a broad range of physiological and biochemical characteristics. Algae are promising source of novel biochemically active compounds like fatty acids, steroids, carotenoids, polysaccharides, lectins, vitamins and phyco-proteins, amino acids, dietary minerals, halogenated compounds, polyketides, toxins and diverse antioxidants. Numerous extraction and analytical techniques were employed to study the chemical composition of algae from different natural sources like marine ecosystem, freshwater habitat and other environmental conditions. A large number of algal products are also find reliability in the food, cosmetic, biochemical and pharmaceutical industries [7–13].

The Indian trans-Himalayan cold desert region of Ladakh represent a valuable source of large number of natural bio-resources. The diverse floral and faunal composition of this remote Himalayan land provides a vast number of natural products beneficial for the armed forces as well as civil population [14]. This high altitude region is one of the most difficult and hostile terrain for human survival due to the extreme environmental factors and scarcity of fresh foods [15]. It is believed that plants growing in high altitude environment produce natural bioactive substances or secondary metabolites, which help them survive under stressful conditions. The high-altitude plant species of Ladakh region have been reported to possess rich medicinal properties and play major role in the traditional systems of medicine since ancient time [16,17] and could be used as prophylactic and therapeutic agent for high altitude maladies and other stress induced disorders [18–20].

The unicellular microalgae in particular have often been considered as a promising, easy and alternative source of useful natural antioxidants [21–27]. The filamentous freshwater green alga *Spirogyra* spp. (family Zygnemataceae) is consumed as food in many Asian countries. They are valuable source of natural bioactive compounds that are widely exploited for antibiotic, antiviral, antioxidant, anti-inflammatory, cytotoxic and other positive biological activities. In recent time, evaluation of chemical composition and biological activity in natural bio-resources is an urgent task for researchers involved in natural product development and medicinal chemistry. A number of chemometric methods are available to determine the bioactive phytochemotypes and determination of biological activities by *in vitro* and *in vivo* assays which equally produce significant results to assess the pharmaceutical, therapeutic and heath promoting properties of natural phytoproducts. These methods have provided an efficient and powerful tool for the quality control, safety and bio-efficacy evaluation of natural products for human consumption [28–37].

Information obtained from different reports revealed that the trans-Himalayan region of Kashmir and Ladakh are a rich source of diverse algal communities [38,39]. We have previously developed a number of herbal products from the high-altitude plants of this region and conducted various studies on medicinal, nutritional and antioxidant potential of these plants and natural products by different biochemical approaches and these products showed prophylactic effects against high-altitude ailments [40–42]. However, there is scarcity of information about the medicinal and therapeutic potential of algae from this mega diversity hotspot, which prompted us to investigate the antioxidant capacities, phenolic content, GC/MS chemometric profile, cytotoxic effect and anti-hypoxic potential of trans-Himalayan alga *Spirogyra porticalis* (Muell.) Cleve from this cold desert region. The present investigation was undertaken with the objective to estimate the *in vitro* antioxidant capacities and phenolic profile of different solvent extracts of *S. porticalis* from Indian trans-Himalaya. The extract showing highest antioxidant capacity and phenolic content was further investigated to determine the chemometric profile using GC/MS hyphenation and the cytotoxic activity was evaluated on human hepatocellular carcinoma HepG2 and colon carcinoma RKO cells. Finally, the anti-hypoxic effect of *S. porticalis* extract was tested on *in vivo* animal system.

## Materials and Methods

### Chemicals and reagents

For antioxidant assays, 1,1-diphenyl-2-picrylhydrazyl (DPPH), 2,2'-azinobis-(3-ethylbenzothiazoline-6-sulfonic acid) diammonium salt (ABTS), 2,4,6-tripyridyl-*s*-triazine (TPTZ), ferrous sulfate (FeSO_4_.7H_2_O), aluminium chloride (AlCl_3_), sodium acetate (C_2_H_3_NaO_2_), sodium carbonate (Na_2_CO_3_), potassium persulfate (K_2_S_2_O_8_), potassium chloride (KCl), ferric chloride (FeCl_3_), sodium nitroprusside {Na_2_[Fe(CN)_5_NO].2H_2_O}, sulfanilic acid (C_6_H_7_NO_3_S), butylated hydroxytoluene (BHT), butylated hydroxyanisole (BHA), ascorbic acid, thiobarbituric acid (TBA), quercetin and catechin were purchased from Sigma-Aldrich (St. Louis, MO, USA). Folin Ciocalteu's phenol reagent, vanillin, hydrochloric acid, sulphuric acid, methanol, n-hexane, acetonitrile, chloroform, ethanol and sodium carbonate from Merck Chemical Supplies (Merck KGaA, Darmstadt, Germany) have been utilized. All the other chemicals used, including solvents, were of analytical grade. Absorbance of the test samples and standards were recorded using spectrophotometer (Spectramax M2^e^, Molecular Devices, Germany). For GC/MS chemical characterization, CHROMASOLV HPLC grade methanol was used and all other chemicals were of analytical grade and purchased from Sigma-Aldrich. For cytotoxicity assays, RPMI medium 1640, penicillin, streptomycin, trypsin-EDTA, sulphorhodamine B, trichloroacetic acid, acetic acid, Tris base and all other analytical grade chemicals were the products from Sigma-Aldrich. For animal studies, the following chemicals *viz*. heparin, 2′,7′-dichlorofluorescein diacetate (DCFHDA), PBS buffer, meta-phosphoric acid, L-glutathione reduced (GSH) and L-glutathione oxidized (GSSG) were also procured from Sigma-Aldrich.

### Ethics statement

The necessary permits for the field studies and collection of algae sample were obtained and it was issued by Dr. B. Balaji (IFS), Divisional Forest Officer, Leh Forest Division, Jammu & Kashmir, India. Experimental animals, along with the necessary facilities requisite for the studies were utilized at our sister laboratory Institute of Nuclear Medicine and Allied Sciences (INMAS), DRDO, New Delhi, India. Animal experimental protocols were approved by the INMAS Institutional Animal Ethical Committee (IAEC/2010, extended up to 31st December, 2013) and conformed to national guidelines Committee for the Purpose of Control and Supervision of Experiments on Animals (CPCSEA) of Government of India on the care and use of laboratory animals. Proper concern was taken to minimize the sufferings of the studied animals during the experiment.

### Algal sample collection

In the fragile microclimate of trans-Himalayan Ladakh region, the average minimum and maximum temperature of Defence Institute of High Altitude Research (DIHAR) campus at 3500 m altitude during 1^st^ March to 30^th^ April, 2011 were −2°C and 11.1°C, which allowed the aggravated growth of *S. porticalis*. The *S. porticalis* sample was collected on 20^th^ April, 2011 from a constructed cemented rectangular pond (size 20×15×2 m, water pH 7.9) of DIHAR, Leh [altitude 3500 m above sea level (ASL), latitude 34°8′16.121″N, longitude 77°34′19.2219″E]. The contamination of other materials like dry waste of some herbs, soil content and earthworms entangled in the algae thallus was carefully avoided from the natural spring habitat (where soil was of sandy desert nature having 56–64% sand content and insignificant amount of organic material) with the help of a small, natural, narrow canal (1 ft depth, 1 ft width and 5–10 cm water level) carrying spring water to the constructed cemented pond. For regular stirring and replacement of old water (except slow moving spring water as inoculum), a fast moving tube-well water was routinely supplied to this pond through the same inlet (cemented canal) and at the same time water is removed by an outlet (outlet cemented canal) daily from 11 a.m. to 4 p.m. The inlet and outlet were entangled with 10 mm and 5 mm sieves respectively to avoid transport of *S. porticalis*. The water depth of the pond was 1–2 ft. Finally, 10 kg wet *S. porticalis* was collected, washed under tap water and air dried in room temperature. The algal material was then preserved in 4% formalin and deposited in the Algal Herbarium, CSIR-National Botanical Research Institute (CSIR-NBRI), Lucknow, India.

### Identification of alga

The algal samples were observed under Leica DM500 research microscope (Leica Microsystems, Wetzlar, Germany) and photographs were taken with attached EC3 digital camera. Relevant publication of Prescott (1951) [43] was referred for the identification of algal taxa. For the taxonomic description of taxa, dimensions were given in micrometer (μm). All measuring scales given for algae photographs were equal to 10 μm. The specimens were preserved in the Algal Herbarium, CSIR-NBRI, Lucknow.

### Algae materials and extraction

*S. porticalis* was air dried at room temperature to constant weight. The dried algal material was ground separately to powder. Finely ground dried sample (50 g) was taken for sequential extraction in four solvent systems with increasing polarity *viz*. n-hexane, acetonitrile, methanol and water by Soxhlet apparatus (Borosil GlassWorks Limited, Worli, Mumbai, India) under the following temperatures such as 20°C for hexane, 30°C for acetonitrile, 40°C for methanol and 45°C for water. Then all extracts were concentrated under reduced pressure at 40°C by cold water circulation using thermostat maintained at 4°C for minimizing the degradation of thermo labile compounds. The water extract was concentrated by sublimation in Freeze Dryer (Christ Alpha 1–4 LSC Freeze Dryer, Martin Christ Freeze Dryers GmbH, Osterode am Harz). All extracts were finally stored at −80°C freezer for further analysis.

### Antioxidant capacities and free radical scavenging assays

**Ferric reducing antioxidant power (FRAP).** The FRAP assay was performed using the method depicted by Ikram and co-workers (2009) with slight alteration [44]. A total of 75 μl extract and 225 μl distilled water were added to 2.25 ml of freshly prepared FRAP reagent [10 parts of 300 mM sodium acetate buffer at pH 3.6, 1 part of 10 mM 2,4,6-tri (2-pyridyl)-*s*-triazine (TPTZ) solution and 1 part of 20 mM FeCl_3_.6H_2_O]. The content was incubated in the dark for 30 min. The increase in absorbance with the formation of colored product (ferrous tripyridyltriazine complex) was measured at 593 nm. The antioxidant capacity of the algal extract was determined based on a calibration curve plotted using FeSO_4_.7H_2_O at a concentration ranging between 0.125 and 2 mM. The calibration equation for FeSO_4_.7H_2_O was y = 0.464x − 0.0114, R² = 0.9996, where x was absorbance at 593 nm and y was concentration of FeSO_4_.7H_2_O (mM). Results were expressed in mM Fe (II)/g of extract.

**ABTS radical scavenging activity.** ABTS assay was performed according to the preciding protocol [45]. The stock solution was prepared by mixing equal volume of 7 mM ABTS solution and 2.45 mM potassium persulfate solution followed by incubation for 12 h at room temperature in the dark to yield a dark-colored solution containing ABTS·^+^ radicals. Working solution was prepared freshly before each assay by diluting the stock solution by mixing of stock solution to 50% methanol for an initial absorbance of 0.700 ± 0.02 at 745 nm, with temperature control set at 30°C. Free radical scavenging activity was assessed by mixing 30 μl of different fractions (different concentration in respective solvents) with 300 μl of ABTS working standard. The decrease in absorbance was measured after 6 min at 30°C. Quercetin was used as positive control. The scavenging activity was estimated based on the percentage of ABTS radicals scavenged by the following formula:

% scavenging = [(A_0_ − As)/A_0_] × 100, where A_0_ was absorbance of control, A_S_ was absorbance of extract. The antioxidant value was expressed as the half maximal inhibitory concentration (IC_50_), the amount of antioxidant required to decrease the initial ABTS concentration by 50%. The IC_50_ value was calculated from the regression equation between sample concentration and rate of inhibition.

### *Inhibition effect on* 1,1-diphenyl-2-picrylhydrazyl radical (DPPH^∙^)

The method suggested by previous investigators was adopted for this purpose with minor modification [46,47]. The 0.1 mM solution of DPPH in methanol was prepared and 500 μl of this solution was reacted with 25 μl of algal extract. The control sample was reacted with 25 μl of solvent instead of the extract. The mixture was incubated at room temperature for 30 min before the shrink in absorbance at 517 nm was recorded. Ascorbic acid was used as positive control. Antioxidant value was expressed as the half maximal inhibitory concentration (IC_50_), the amount of antioxidant required to decrease the initial DPPH concentration by 50%. The IC_50_ values were calculated as described previously.

**β-carotene-linoleic acid bleaching assay.** The method described by Dapkevicius et al. (1998) [48] was used for β-carotene-linoleic acid bleaching assay. A stock solution of β-carotene and linoleic acid was prepared as follows: 0.5 mg of β-carotene was dissolved in 1 ml of chloroform, and 25 μl of linoleic acid with 200 mg of Tween 20 were added. After chloroform evaporation under vacuum, 100 ml of distilled water was added to the residue. An aliquot of 500 μl of extract (in different concentration) was pipetted into separated test tubes and 5 ml of the previous mixture was added. The test tubes were incubated for 2 h at 50°C together with the control sample. The absorbance was measured at 470 nm at the beginning (t = 0 min) and after the experiment (t = 120 min). BHT was used as positive control. The antioxidant capacity was calculated as percentage inhibition of oxidation (% AA) using the following equation:

% AA = [1 –(Abs_s_^0^—Abs_s_^120^)/(Abs_c_^0^—Abs_c_^120^)] × 100, where Abs_s_^0^ was absorbance of sample at t_0_, Abs_s_^120^ was absorbance of sample at t_120_, Abs_c_^0^ was absorbance of control at t_0_, Abs_c_^120^ was absorbance of control at t_120_. The IC_50_ values were then calculated by aforementioned method.

**Nitric oxide (NO) scavenging activity.** The previously published method was used to assay the scavenging activity of extracts on nitric oxide (NO) with minor modification [49]. The reaction solution (100 μl) containing 10 mM sodium nitroprusside in phosphate buffer saline (PBS, pH 7.0) was reacted with 10 μl extract (of different concentration). The reaction mixture was then incubation at 37°C for 1 h. Thereafter 50 μl aliquot was mixed with 50 μl of Griess reagent and the absorbance at 540 nm was measured. Percent inhibition of NO produced was calculated by comparing with the absorbance value of the negative control (10 mM sodium nitroprusside and PBS). BHA was used as the positive control. Finally, the IC_50_ value was calculated.

**Lipid peroxidation assay.** A modified thiobarbituric acid reactive species (TBARS) assay [50] was employed to measure the lipid peroxide formed using egg yolk homogenate as lipid rich media [51]. Egg homogenate (0.5 ml of 10%, v/v) and 0.1 ml of extract were added to a test tube and made up to 1 ml with distilled water. After that, 0.05 ml of FeSO_4_ (0.07 M) was added to induce lipid peroxidation and the mixture was incubated for 30 min. Then, 1.5 ml of 20% acetic acid (pH 3.5) and 1.5 ml of 0.8% (w/v) thiobarbituric acid in 1.1% sodium dodecyl sulphate were added and the resulting mixture was vortexed and then heated at 95°C for 60 min. After cooling, 5 ml of butan-1-ol was added to each tube and centrifuged at 3,000 rpm for 10 min. The absorbance of the organic upper layer was measured at 532 nm. Inhibition of lipid peroxidation (%) by the extract was calculated as [(1−E)/C] x 100, where C was absorbance of the fully oxidized control and E was absorbance in presence of extract. The IC_50_ value was calculated as depicted previously.

### Total proanthocyanidin, flavonoid and polyphenolic content

**Total polyphenol content (TPC).** TPC was determined by Folin Ciocalteu’s colorimetric method as described by previous investigators [52]. Extract solution (10 μl) was mixed with 20 μl of 10% Folin Ciocalteu’s reagent and 200 μl of H_2_O, following incubation at room temperature for 3 min. After that 100 μl of sodium carbonate (20%, w/v) was added to the reaction mixture. The content was incubated at room temperature in the dark for 30 min and absorbance was measured at 765 nm. Estimation of total polyphenol was carried out on the basis of calibration curve: y = 0.0104x − 0.0589, R² = 0.9978, where x was absorbance and y was gallic acid equivalent (GAE) at a final concentration of 100 μg/ml. The results were expressed in GAE (mg/g of extract).

**Total flavonoid content (TFC).** TFC was analyzed with the method of Ordonez et al. (2006) [53]. Briefly, 100 μl of sample and 2% AlCl_3_ in ethanol solution were allowed to react and the reaction mixture was incubated for 1 h at room temperature and the absorbance was finally measured at 420 nm. Total flavonoid content was calculated as quercetin equivalent (QE) in mg/g of extract using the following equation based on the calibration curve: y = 0.0235x − 0.1803, R² = 0.9994, where *x* was absorbance and y was QE (mg/g of extract) at final concentration 100 μg/ml.

**Total proanthocyanidins content (TPAC).** The TPAC content was determined according to the method of Sun et al. (1998) [54] with slight modification. A volume of 0.5 ml of extract solution was allowed to react with 3 ml of 4% vanillin-methanol solution and 1.5 ml hydrochloric acid. Then the mixture was allowed to stand for 15 min. The absorbance was measured at 500 nm at 30°C. Total proanthocyanidin content was calculated as catechin equivalent (CE) in mg/g of extract using the following equation based on the calibration curve: y = 0.0015x − 0.0209, R² = 0.9975, where x was absorbance and y was CE (mg/g of extract). Extract samples were evaluated at a final concentration of 100 μg/ml.

### GC/MS analysis

**Preparation of sample for GC/MS analysis.** The concentrated methanol extract (25 mg) was dissolved in 25 ml of the solvent, vortexed properly and filtered through 0.22 μm syringe filter (Millipore Corp., Bedford, MA, USA). For requisite analysis, one microlitre aliquot of the sample solution was then injected into the GC/MS MS system.

**Instrumentation and chromatographic conditions.** Thermo Finnigan PolarisQ Ion Trap GC/MS MS system comprising of an AS2000 liquid autosampler (Thermo Finnigan, Thermo Electron Corporation, Austin, TX, USA) was used for GC/MS analysis. As per our previous work, the peaks in the chromatogram were identified on the basis of their mass spectra [40,55]. The gas chromatograph was interfaced to a mass spectrometer instrument utilizing the following conditions *viz*. Durabond DB-5 ms column (30 m× 0.25 mm× 0.25 μm), operating in electron impact [electron ionisation positive (EI+)] mode at 70 eV, helium (99.999%) as carrier gas at a constant flow of 1 ml/min and an injection volume of 0.5 EI (split ratio of 10:1), injector temperature 280°C and transfer line temperature 300°C. The oven temperature of the system was programmed from 50°C (isothermal for 2 min), with gradual increase in steps of 10°C/min, to 300°C. Mass spectra were taken at 70 eV, a scan interval of 0.5 s and full mass scan range from 25 m/z to 1000 m/z. Finnigan Xcalibur data acquisition and processing software version 2.0 (ThermoQuest, LC and LC/MS Division, San Jose, California, USA) was used for data acquisition.

**Identification of components.** NIST/EPA/NIH Mass Spectral Database (NIST11), with NIST MS search program v.2.0 g [National Institute Standard and Technology (NIST), Scientific Instrument services, Inc., NJ, USA] was utilized for the interpretation GC/MS mass spectrum. Finally, name, molecular weight and structure of the components of the test material were determined.

### Cytotoxic effects of *S. porticalis* methanol extract on carcinoma cells

**Cell cultures.** Two cancerous cell lines namely human hepatocellular cancer (HepG2) and colon cancer (RKO) cell lines were procured from American Type Cell Culture (ATCC), United States of America (USA). The RPMI medium 1640 (supplemented with 10% heat-inactivated foetal bovine serum, 100 units/ml of penicillin and 100 μg/ml of streptomycin, pH 7.4) was used to maintain the cells at 37°C in 5% CO_2_ and 95–100% humidified air atmosphere.

**Morphological determination of cell toxicity.** Cells growing logarithmically were seeded in 96 flat-well plate (HepG2 cells 3,000/well and RKO cells 3,000/well) and incubated for 24 h to have a partial monolayer. Cells were treated with 29.26, 43.90, 65.84, 98.77, 148.15, 222.22 and 333.33 μg/ml of *S. porticalis* methanol extract dissolved in RPMI 1640 medium and subsequently incubated at 37°C in a 5% CO_2_ and 95–100% humidified air atmosphere (Touch 190S, LEEC, Nottingham, UK). Microscopic assessment was performed under 10X eyepiece and 10X objective lenses with inverted phase contrast microscope (Dewinter Optical, Inc., Delhi, India) and the changes in cellular morphology following different treatments were recorded at 24, 48 and 72 h of time intervals.

**Sulphorhodamine B (SRB) assay for cytotoxicity screening.** The Sulphorhodamine B (SRB assay) was used for determining cellular growth based on the SRB uptake by cellular protein. The SRB assay for colorimetric determination of cytotoxicity of *S. porticalis* methanol extract was performed [56,57]. The cells were harvested with 0.5% trypsin-EDTA. Then just before T_0_ time (T_0_ = zero time after extract inoculation in the cell culture) and just after T_t_ time (T_t_ = after 72 h of extract inoculation in cell culture) of plate incubation at 37°C in 5% CO_2_, in humidified incubator (95–100% humidity), the SRB assay was performed. The medium was removed at both time points and the cells were fixed with trichloroacetic acid (20%, w/v) at 4°C for 1 h, stained for 30 min with SRB (0.4%, w/v) dissolved in 1% acetic acid for 30 min, and washed four times with 1% acetic acid. The protein-bound dye was made solubilised with 10 mmol/L tris base, pH 10.5. The absorbance of each well was read at 565 nm using spectrophotometer (Spectramax M2^e^, Molecular Devices, Germany).

Percentage growth (PG) at each concentration level was calculated as:

% growth = [(T_t_ − T_0_)/(C − T_0_)] × 100 for concentrations in which T_t_ ≥ T_0_ and

% growth = [(T_t_ − T_0_)/ T_0_] × 100 for concentrations in which T_t_ < T_0_

The 50% growth inhibition concentration (GI_50_, μg/ml) was determined as the concentration where {(T_t_ − T_0_)/(C − T_0_)} × 100 = 50. Total growth inhibition concentration (TGI, μg/ml) was measured as the concentration where {(T_t_ − T_0_)/(C − T_0_)} × 100 = 0 = PG. Lethal concentration that kills 50% of the cells (LC_50_, μg/ml) was calculated as the concentration level where {(T_t_ − T_0_)/ T_0_} × 100 = −50. These parameters were determined in accordance with previous reports [58–60]. Here, T_0_ = growth in the presence of *S. porticalis* extract at time zero, C = control growth and T_t_ = growth in the presence of *S. porticalis* extract after 72 h.

### Effect of *S. porticalis* methanol extract on hypobaric hypoxia induced oxidative stress

**Administration of algal extract.** Vacuum dried methanol extract was dissolved in distilled water to obtain the desired dose of 200 mg/kg body weight of the animal and administered to rats through oral route in a maximum volume of 1 ml through gastric cannula.

**Animals.** The animal study experimental protocols were approved by the Institutional Ethics Committee and proper concern was taken to minimize the sufferings of the studied animals during the experiment. Adult male Sprague-Dawley rats weighing 220 ± 10 g were used for the study. The animals were housed in hygienic conditions with day and night cycle of 12 h each with temperature maintained at 25 ± 2°C and humidity at 65 ± 5%.

**Altitude simulation.** A specially designed animal decompression chamber was used to expose the animals to simulated altitude of 7,600 m (25,000 ft, 282 mm Hg) for hypobaric hypoxia environment. Here altitude was maintained by reducing the barometric pressure and temperature and humidity were controlled precisely. Continuous hypoxic exposure was provided for the stipulated period with a 10–15 min interval each day for drug administration, food and water replenishment and for cage changing purpose. Continuous flushing of fresh air at a rate of 8 l/min was maintained for preventing the accumulation of carbon dioxide within the chamber. The temperature and humidity in the hypobaric chamber were kept at 32 ± 2°C and 63 ± 3% respectively. The ascending and descending rate to hypobaric condition was maintained at 300 m/min. The normoxic animals were kept under similar temperature and humidity conditions in a separate chamber maintained at ambient barometric pressure. A 12 h day and night cycle was maintained in both chambers during the period of exposure.

**Experimental design.** Male adult Sprague-Dawley rats (n = 5/group) were randomly divided into three groups: *Group I*: served as control group (normoxia group with 0 day exposure), *Group II*: served as hypoxia + vehicle group in which rats were exposed to a simulated altitude of 7,600 m and orally administrated with 1 ml of vehicle (distilled water) once in a day for 7 days, *Group III*: served as hypoxia + drug treated group in which rats were exposed to 7,600 m simulated altitude and orally administrated with 1 ml of algal extract once in a day for 7 days.

Rats were housed one animal per cage for all study groups for 2 days habituation before the initiation of experimental procedures. The hypoxia groups were allowed to habituate and acclimatize to the experimental conditions for 2 days before the initiation of experimental procedures. Body weight, food consumption and water intake of rats were measured every day and recorded. Food and water were freely available to the animals during the experiment. One millilitre of distilled water was used as vehicle. The ARRIVE guidelines were followed for reporting the animal research investigation [61]. On completion of the stipulated period of hypoxic exposure, fasting experiments were performed for 24 h and animals were sacrificed at 9.30 a.m. Rats were euthanized by CO_2_ asphyxiation followed by cervical dislocation [62]. Then blood samples were obtained from orbital sinus in heparin coated blood collection vial [63].

**ROS generation.** Following the stipulated period of exposure, serum sample was taken from animals and sample homogenate was prepared in 0.15 M KCl. The free radicals were estimated spectrofluorimetrically with 25 μl of sample homogenate using DCFHDA as described by LeBel et al. (1990) [64] with slight changes. In brief, 1.494 ml of 0.1 M PBS (pH 7.4) was added to 25 μl of the sample followed by addition of 6 μl of 1.25 mM DCFHDA. The sample was then incubated for 15 min at 37°C in dark and the fluorescence was measured at 488 nm excitation and 525 nm emission. The readings thus obtained were converted to fluorescent units/mg protein by calculating the protein present in 25 μl of each sample from a standard curve.

**GSH and GSSG.** Reduced glutathione (GSH) and oxidized glutathione (GSSG) levels were measured fluorimetrically from the sample homogenate according to the procedure followed by Hissin and Hilf (1976) [65] with minor modifications. In brief, to 250 μl of the sample homogenate was added to an equal volume of 10% meta-phosphoric acid, followed by centrifugation at 10,000 g for 30 min at 48°C. The supernatant thus obtained was used for estimation of GSH and GSSG spectrofluorimetrically at 350 nm excitation and 420 nm emission.

### Statistical analysis

All experiments were performed in triplicate. The mean values of three individual observations were determined and the standard deviation (SD) was calculated followed by one way analysis of variance (ANOVA) to determine the level of significance among means. The values are represented as mean ± SD. Statistical difference between groups was calculated using one-way analysis of variance followed by the Neumann-Keuls test for post hoc analysis. The *p* value < 0.05 was considered to be statistically significant.

## Results

### Morphological identification of alga

**Fig 1:**
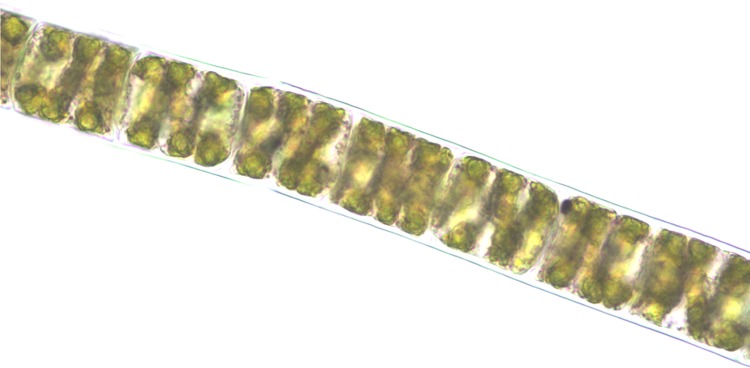
Morphology of *S. porticalis* under Leica DM500 research microscope.

The morphology of algal specimen was studied carefully and the specimen was identified as ***Spirogyra porticalis* (Muell.) Cleve 1868**. The taxonomic description and identification characters were as follows: filament of rather shout cells, 40–50 μm in diameter, 68–200 μm long, with plane end walls; chloroplast solitary, making 3 to 4 turns; conjugation scalariform by tubes from both gametangia; fertile cells sometimes inflated; zygospores ovate to subglobose-ovate; median spore wall smooth and yellow, 38–50 μm in diameter, 50–83 μm long (Fig. 1); common in shallow water of lakes, swamps and roadside ditches [43]. *S. porticalis* showed its appearance in the second week of March, 2011 and found till last week of April to first week of May, 2011 in the spring water and cemented pond in DIHAR.

### Antioxidant capacities and phenolic content

In the FRAP assay, methanol extract showed highest (171.49 mM Fe (II)/g extract) and hexane extract exhibited lowest (18.91 mM Fe (II)/g extract) antioxidant capacities. In ABTS assay, methanol extract showed highest antioxidant capacity (IC_50_ 3.48 mg/ml) and water extract showed lowest antioxidant capacity (IC_50_ 37.71 mg/ml). In β-carotene-linoleic acid assay, methanol extract again showed highest antioxidant capacity (IC_50_ 0.20 mg/ml) and acetonitrile extract showed lowest antioxidant capacity (IC_50_ 11.23 mg/ml). In DPPH assay, methanol extract showed highest antioxidant capacity with an IC_50_ value of 0.52 mg/ml and hexane extract showed lowest antioxidant capacity with 10.94 mg/ml IC_50_ value. In nitric oxide assay, methanol extract exhibited highest antioxidant capacity (IC_50_ 4.31 mg/ml) and acetonitrile extract showed lowest antioxidant capacity (IC_50_ 61.11 mg/ml). In Lipid peroxidation assay, methanol extract was found to possess highest antioxidant capacity (IC_50_ 0.06 mg/ml) and water extract showed lowest antioxidant capacity (IC_50_ 1.38 mg/ml). In all antioxidant assays, the methanol extract of *S. porticalis* was found to exhibit significantly higher (*p*<0.05) antioxidant capacities than all other studied extracts (Table 1).

**Table 1:** Antioxidant capacities of *S. porticalis* extracts^a^.

Antioxidant capacity	SWE	SME	SAE	SHE
FRAP (mM Fe (II)/g extract)	32.69 ± 3.86	171.49 ± 19.25*	137.70 ± 11.16* ^#^	18.91 ± 1.46* ^#^ ^$^
ABTS IC_50_ (mg/ml)	37.71 ± 0.09	3.48 ± 0.11*	24.73 ± 0.09* ^#^	4.68 ± 0.11* ^#^ ^$^
β-carotene IC_50_ (mg/ml)	0.43 ± 0.01	0.20 ± 0.00*	11.23 ± 0.10* ^#^	0.33 ± 0.02* ^#^ ^$^
DPPH IC_50_ (mg/ml)	8.06 ± 0.10	0.52 ± 0.01*	5.23 ± 0.11* ^#^	10.94 ± 0.08* ^#^ ^$^
NO IC_50_ (mg/ml)	9.54 ± 0.09	4.31 ± 0.11*	61.11 ± 0.11* ^#^	31.47 ± 0.08* ^#^ ^$^
Lipid peroxidation IC_50_ (mg/ml)	1.38 ± 0.11	0.06 ± 0.01*	0.42 ± 0.00* ^#^	0.33 ± 0.01* ^#^ ^$^

^a^Mean ± SD of three replicates.

SWE: *S. porticalis* water extract, SME: *S. porticalis* methanol extract, SAE: *S. porticalis* acetonitrile extract, SHE: *S. porticalis* n-hexane extract.

*p*<0.05

*compared with SWE

^#^compared with SME

^$^compared with SAE

Total phenolic content was predominant in methanol extract (96.70 mg GAE/g extract) and lowest in water extract (14.90 mg GAE/ g extract). Similarly, total flavonoid content rendered highest value in methanol extract (37.59 mg QE/g extract) and lowest in water extract (13.82 mg QE/g extract). Total proanthocyanidin content was also highest in methanol extract (122.55 mg CE/g extract) and minimum in water extract (25.51 mg CE/g extract). The methanol extract of *S. porticalis* was found to exhibit significantly higher (*p*<0.05) total phenolics than all other extracts (Table 2).

**Table 2:** Total phenolic (TPC), flavanoid (TFC) and proanthocyanidin content (TPAC) in *S. porticalis* extracts from Indian trans-Himalaya^a^.

Phytochemicals	SWE	SME	SAE	SHE
TPC (mg GAE/ g)	14.90 ± 1.26	96.70 ± 9.64*	21.42 ± 1.07* ^#^	23.72 ± 2.5* ^#^
TFC (mg QE/g)	13.82 ± 1.36	37.59 ± 2.80*	34.76 ± 2.99*	35.30 ± 3.02*
TPAC (mg CE/g)	25.51 ± 2.25	122.55 ± 10.12*	78.36 ± 5.78* ^#^	94.79 ± 7.74* ^#^ ^$^

^a^Mean ± SD of three replicates.

SWE: *S. porticalis* water extract, SME: *S. porticalis* methanol extract, SAE: *S. porticalis* acetonitrile extract, SHE: *S. porticalis* n-hexane extract.

*p*<0.05

*compared with SWE

^#^compared with SME

^$^compared with SAE

### GC/MS chemometric profile of methanol extract

**Fig 2:**
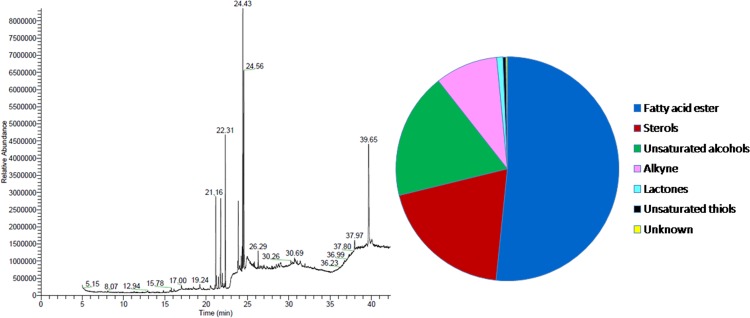
GC/MS chemometric profile of *S. porticalis* methanol extract.

As described previously, in course of investigation for the preliminary screening with respect to antioxidant capacities and total phenolic content of different extracts of *S. porticalis*, the highest antioxidant capacities as well as the total phenolic content was achieved by the methanol extract. Henceforth, it was further evaluated for GC/MS chemometric profiling. GC/MS chromatograms of methanol extract of *S. porticalis* as per aforementioned experimental procedure showed various peaks indicating the presence of different cluster of phytochemotypes in the extract (Fig. 2).

The methanol extract of *S. porticalis* revealed the presence of thirteen different chemotypes which were characterized and identified by comparison of their mass fragmentation patterns with the similar compounds present in the NIST database. Of these thirteen chemotypes, ethyl linoleolate (21.32%), stigmasta-5,24(28)-dien-3-ol, (3*β*) (19.55%), 3,7,11,15-tetramethyl-2-hexadecen-1-ol (18.22%), methyl palmitate (hexadecanoic acid, methyl ester) (12.01%), 5,8,11,14,17-eicosapentaenoic acid, methyl ester, (all-Z) (9.82%), 8-hexadecyne (8.05%), and 4,7,10,13,16,19-docosahexaenoic acid, methyl ester, (all-Z) (7.26%) were found to be the major constituents. In addition, ethyl-5,8,11,14,17-icosapentaenoate (1.23%), 3-heptadecen-5-yne, (Z) (1.02%), (-)-loliolide (0.55%), 1,5-hexadien-3-ol, 3-methyl-6-(methylthio)-1-(2,6,6-trimethyl-1-cyclohexen-1yl) (0.43%), dihydroactinidiolide (0.34%) and an unknown compound (0.20%) were detected in trace amount (Table 3, Fig. 2). The identified compounds were further investigated for their biological activities and most of them were found to possess diverse range of positive pharmacological and therapeutic functions [66–85] (Table 3).

**Table 3:** Algal chemotypes identified in methanol extract of *S. porticalis* by GC/MS.

**S. No.**	**Peak RT (min)**	**Peak area**	**Peak area (%)**	**Compound detected**	**Mol. Formula**	**Mol. Wt.**	**CAS No**	
1	15.78	156723	0.20	Unknown	-	-	-	-
2	17.00	257237	0.34	Dihydroactinidiolide	C_11_H_16_O_2_	180	15356–74–8	Antibacterial; antiproliferative; cytotoxic
3	19.24	335417	0.43	1,5-hexadien-3-ol, 3-methyl-6-(methylthio)-1-(2,6,6-trimethyl-1-cyclohexen-1-yl)	C_17_H_28_OS	280	97369–78–3	-
4	20.53	422013	0.55	Loliolide	C_11_H_16_O_3_	196	5989–02–6	Strong anti-repellent; antioxidant; protective effects against H_2_O_2_-induced cell damage
5	21.16	6137430	8.05	8-Hexadecyne	C_16_H_30_	222	19781–86–3	Used in the preparation of fatty alcohols with potential for application in commercial products like as bio-diesel, lubricants, greases and cosmetics
6	21.76	7509928	9.82	5,8,11,14,17-Eicosapentaenoic acid, methyl ester, (all-Z)-	C_21_H_32_O_2_	316	2734–47–6	Hypolipidemic action; lipid-lowering effect; enhancing effect on hepatic biliary secretion; anti-arteriosclerotic
7	21.95	791387	1.02	3-Heptadecen-5-yne, (Z)-	C_17_H_30_	234	74744–55–1	-
8	22.31	9034647	12.01	Hexadecanoic acid, methyl ester	C_17_H_34_O_2_	270	112–39–0	Antioxidant; nematicide; pesticide; lubricant; antiandrogenic; flavor, hemolytic 5-alpha reductase inhibitor; hypocholesterolemic
9	23.88	5526076	7.26	4,7,10,13,16,19-Docosahexaenoic acid, methyl ester, (all-Z)-	C_23_H_34_O_2_	342	2566–90–7	-
10	24.43	16103379	21.32	Ethyl Linoleolate	C_20_H_36_O_2_	308	544–35–4	Antioxidant; lightens UV-induced skin pigmentation; anti-inflammatory; competitive inhibitor of prostaglandin production; wounds healing properties; effective anti-acne agent
11	24.56	13175492	18.22	3,7,11,15-Tetramethyl-2-hexadecen-1-ol	C_20_H_40_O	296	102608–53–7	Used in manufacturing synthetic vitamins E and K; an ingredient of fragrances; end applications include soap, detergent, beauty care product, household product; cancer preventive properties
12	26.29	945579	1.23	Ethyl-5,8,11,14,17-icosapentaenoate	C_22_H_34_O_2_	330	84494–70–2	It has lipid-lowering or hypolipidemic effects, provides protection against atherosclerosis and its complications, cardio-protective functions
13	39.67	14709007	19.55	Stigmasta-5,24(28)-dien-3-ol, (3*β*)-	C_29_H_48_O	412	18472–36–1	Androgenic; angiogenic; anorexic; antiadenomic; antiandrogenic; antibacterial; anticancer; antiedemic; antiestrogenic; antifeedant; antifertility; antigonadotrophic; antihyperlipoproteinaemic; anti-inflammatory, antileukemic

### Cytotoxic effects of methanol extract

**Fig 3:**
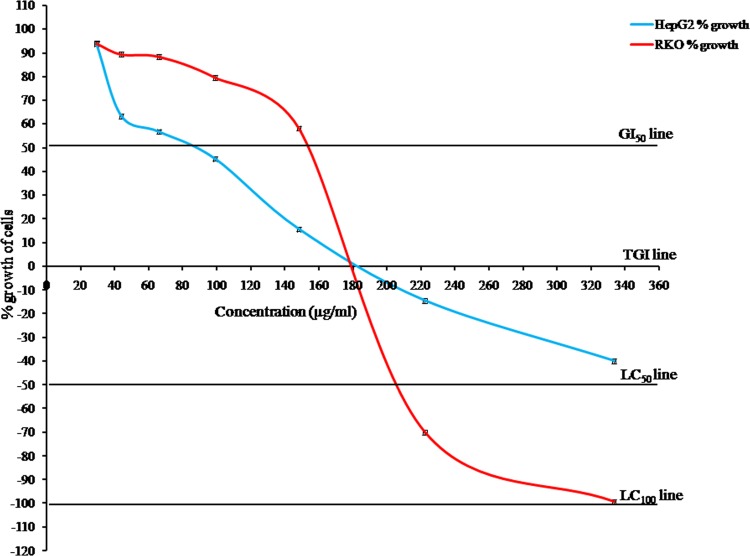
Cytotoxic effect of *S. porticalis* methanol extract on HepG2 and RKO cells.

**Cell cytotoxicity by SRB assay.** In HepG2 cells, the methanol extract was found to be non-toxic at concentration level 43.90 μg/ml (63.32% growth at 43.90 μg/ml extract concentration after 72 h). The GI_50_ and TGI of this extract were 86 μg/ml and 183 μg/ml respectively for HepG2 cells. The % growth at highest concentration level (333.33 μg/ml) was recorded as −39.91. In case of RKO cells, the methanol extract was non-toxic at concentration level of 98.77 μg/ml (79.56% growth at 98.77 μg/ml extract concentration after 72 h). The methanol extract of the alga was found to cross the LC_99_, LC_50_, TGI and GI_50_ line at 333, 205, 179 and 154 μg/ml respectively for RKO cells. All these results have been depicted in Fig. 3 and Table 4.

**Table 4:** Cytotoxicity of methanol extract of *S. porticalis* against HepG2 and RKO cells.

Concentration (μg/ml)	Control (C)			HepG2 cells			RKO cells		
	After 24 h	After 48 h	After 72 h	After 24 h	After 48 h	After 72 h	After 24 h	After 48 h	After 72 h
333.33	nt	nt	nt	d	d	d	d	d	d
222.22	nt	nt	nt	dfp	dep	dep	gapf	dsp	dfp
148.15	nt	nt	nt	nt	ga	ga	nt	gafp	nt
98.77	nt	nt	nt	nt	nt	nt	nt	nt	nt
65.84	nt	nt	nt	nt	nt	nt	nt	nt	nt
43.90	nt	nt	nt	nt	nt	nt	nt	nt	nt
29.26	nt	nt	nt	nt	nt	nt	nt	nt	nt

nt: non-toxic, ga: growth arrested, gafp: growth arrested 50%, dfp: death 50%, dsp: death 70%, dep: death 80%, d: dead

**Fig 4:**
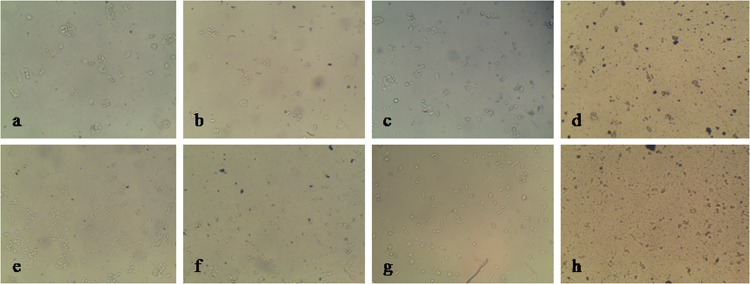
Microscopic images (10×10X) showing cell growth and morphology after 24 h treatment of *S. porticalis* extract in HepG2 and RKO cancer cells. **a**. HepG2 cells with good growth, **b.** HepG2 cells with partially arrested or 50% arrested growth, **c.** HepG2 cells with arrested growth, **d.** HepG2 dead cells, **e.** RKO cells with good growth, **f.** RKO cells with partially arrested or 50% arrested growth, **g.** RKO cells with arrested growth, **h.** RKO dead cells.

**Cell morphology by microscopic analysis.** In HepG2 cells, after 24, 48 and 72 h of extract treatment, it was found to be non-toxic at concentration level 148.15, 98.77 and 98.77 μg/ml respectively whereas the extract showed dead cells at 222.22 and 333.33 μg/ml concentration after 24, 48 and 72 h along with stressed condition at concentration 148.15 μg/ml after 48 and 72 h. In morphological visualization after 24, 48 and 72 h of methanol extract treatment, it was observed as non-toxic at concentration level of 148.15, 98.77 and 148.15 μg/ml respectively for RKO cells. The extract showed dead cells at 222.22 and 333.33 μg/ml concentration after 24, 48 and 72 h (for concentration 222.22 μg/ml after 24 h treatment there was partially arrested RKO cells). All these results have been depicted in Fig. 4.

### Effect of algae methanol extract following hypoxia induced oxidative stress

**Fig 5:**
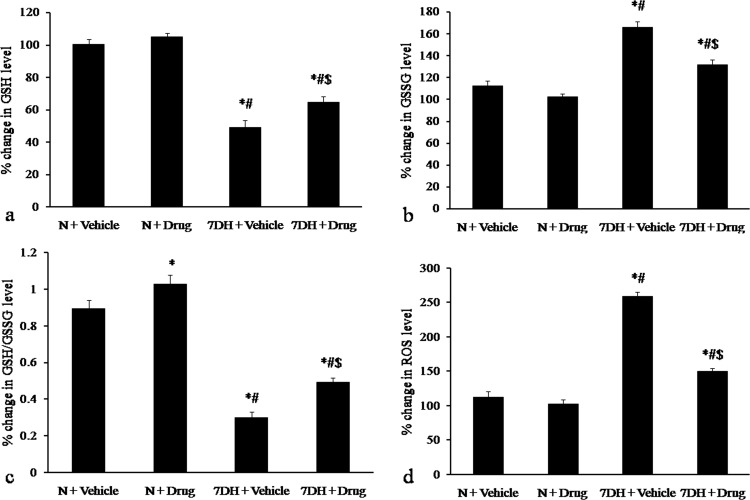
Changes in serum GSH, GSSG, GSH/GSSG and ROS level following administration of *S. porticalis* methanol extract during exposure to hypobaric hypoxia. **a.** Changes in reduced glutathione level, **b.** Changes in the level of oxidised glutathione, **c.** Changes in GSH/GSSG level, **d.** Changes in reactive oxygen species generation. * denotes *p*≤0.05 when compared to normoxia, # denotes *p*≤0.05 when compared to normoxia + vehicle, $ denotes *p*≤0.05 when compared to hypoxia + vehicle.

**GSH, GSSG and GSH/GSSG level.** GSH level in rat blood decreased significantly following exposure to hypobaric hypoxia for 7 days compared to the normoxic group. *S. porticalis* methanol extract administration during hypoxic exposure significantly increased GSH level as compared to the vehicle treated animals of 7 days hypoxic group. However, the GSH level in extract treated group was significantly lower when compared with the normoxic group (Fig. 5A).

GSSG level in rat blood increased significantly following 7 days hypoxic exposure in comparison with the normoxic group. Administration of *S. porticalis* methanol extract to animals during hypoxic exposure significantly caused decrease in the GSSG level as compared to 7 days vehicle treated hypoxic group. However, the GSSG level in extract treated group was significantly higher when compared with the normoxic group (Fig. 5B).

From our results it was clearly observed that there was significant decrease in the GSH level with a concomitant increase in GSSG on animals with 7 days of exposure to hypobaric hypoxia when compared to the normoxic group. The GSH/GSSG value also increased significantly in the 7 days hypoxic animals treated with extract in comparison with the vehicle treated animals of 7 days hypoxic group. However, GSH/GSSG values were found to be reduced significantly in both 7 days hypoxic rats treated with vehicle and extract when compared with the normoxic rats (Fig. 5C).

**Estimation of ROS.** There was a significant increase in ROS generation in rats following 7 day of hypoxia when compared with the normoxic group. The *S. porticalis* extract treated hypoxic rats showed significant reduction in ROS generation in comparison to the hypoxic vehicle group. However, the free radical generation in *S. porticalis* extract treated hypoxic group was significantly higher when compared with the normoxic group (Fig. 5D).

## Discussion

Algae are exposed to very high light intensity and oxygen concentration during their life cycle and growth that support generation of free radicals and other potent oxidizers. The resistance of algal cellular and structural components against this oxidative challenge indicates that the intracellular bioactive antioxidants may be responsible for the preventive role as an intrinsic mechanism for sustenance [86]. In the trans-Himalayan cold desert, high ultraviolet (UV) and infra-red (IR) irradiation in the thin atmosphere, along with freezing temperature (chilling stress) cause unique abiotic stress for algal survival and this atmospheric stressor could be helpful for the production of various antioxidant compounds. Research investigations by Duval et al. (2000) [87] and Kovácik et al. (2010) [88] revealed that the phenolic substances in algal biomass increased upon exposure to UV, which had a positive correlation to its antioxidant properties.

*S. porticalis* is a group of microalga belonging to the family Zygnemataceae. Based on the diverse target compounds, growth rate, cultivation simplicity, greater biodiversity and other allied factors, the micro-algal community represent a hitherto untapped resource of natural antioxidants [21]. The benefit of antioxidant consumption is generally associated with consuming different plants, fruits, vegetables and other related natural food sources but little emphasis has been put forward towards microalgae consumption for corresponding health effects. Although a number of previous reports are available on the ethnobotany, medicinal properties, antioxidant capacity, bioactive chemical composition, cytotoxicity etc. of trans-Himalayan medicinal plants and lichens [40–42,89–91], no scientific investigation is documented on the algal species having medicinal and therapeutic effect and bioactive phytochemicals from this region as far as our information goes.

The antioxidant capacity of plant extracts have been recognized by different redox reactions by several workers such as radical scavenging and inhibitory mechanism, prevention of chain initiation, binding of transition metal ion catalysts, reductive ability etc. [92–94] and it also depends on the hydrophilic or lipophilic nature of the components present in the extracts [40]. Therefore, in order to study the antioxidant processes, the present investigation utilized a range of solvents with varied polarity to access and better understand the trend of antioxidant properties of the different solvent extracts in various antioxidant assay system. We have also monitored the total content of different phytochemicals like proanthocyanidin, polyphenol, flavonoid present in the different extracts in order to determine the relation of phenolic content with the antioxidant capacities. Recent reports by other researchers have described a significant positive correlation between the antioxidant capacities with total phenolic content in microalgal species [21,23], which give support to our results.

Polyphenol, flavonoid, flavonol, proanthocyanidin, alkaloid, terpenoid, steroid, sterol etc. are the class of bioactive compounds of botanical origin that possess strong antioxidant and therapeutic potential [95] and these secondary metabolites are the crucial regulators of plant growth and development in stressful and unfavorable environment. The harsh climatic conditions such as severe cold, aridity, water scarcity, UV radiation etc. in the Indian trans-Himalayan cold desert result in the production and accumulation of anti-stress compounds and secondary metabolites that are known for their antioxidant, medicinal and therapeutic properties and these bioactive compounds could be responsible for the biological activities [87,96,97]. Our results were in concordance with previous investigations where *Spirogyra* extracts showed positive biological activities in terms of antioxidant capacity, antimicrobial activity, antihemolytic activity, antihyperglycemic and antihyperlipidemic properties and renoprotective effect and these activities were attributed to diverse group of bioactive phytochemicals present in this alga [36,37,98–101]. The methanol extract exhibited significantly higher antioxidant capacities and total polyphenol content as compared to n-hexane, acetonitrile and water extracts. Henceforth, we aimed towards the chemometric profiling of the methanol extract by GC/MS technique for its detail chemical characterization. In this investigation our primary objective was the screening of *S. porticalis* extracts on the basis of antioxidant capacities, radical scavenging activities and phenolic profile. The extract showing highest antioxidant potential and phenolic attributes were taken for evaluation of biological activity. Therefore, thorough chemometric profiling was necessary to identify the major bioactive phytochemotypes present in the methanol extract possessing antioxidant properties. To validate the traditional uses, pharmacological actions and therapeutic potential of medicinal plants and other botanicals, metabolic profiling by high-throughput technologies have become an important strategy in medicinal chemistry and natural product research [102–104]; however, GC/MS remains a technique of choice for characterization of volatile compounds present in natural products. The GC/MS chemometric fingerprinting of methanol extract of *S. porticalis* showed thirteen bioactive chemotypes having putative phyto-pharmaceutical importance. The major phytochemical groups namely fatty acid esters, sterols, unsaturated alcohols, alkynes etc. were found to be the dominant components and these are well known for their positive biological functions and could be responsible for the high antioxidant capacities observed in methanol extract of *S. porticalis*.

From the present study, it was established that the methanol extract of *S. porticalis* have promising antioxidant potential and diverse bioactive phytochemicals and it can be utilized as a potential natural antioxidant for various purposes. However, the cytoprotective effects of the algal extract in cell culture system is required to be investigated for further validation of the prophylactic and therapeutic potential and *in vitro* antioxidant capacity of this natural bioresource [105]. We therefore extended our investigation towards the evaluation of cytotoxicity of the methanol extract of *S. porticalis* on human hepatocellular carcinoma HepG2 and colon carcinoma RKO cells.

From the cytotoxicity tests in both cell lines, our results have clearly described the fact that GI_50_ of *S. porticalis* methanol extract was 86 μg/ml for HepG2 cells, which was well below 100 μg/ml and according to the NCI (National Cancer Institute) guideline, this extract could act as a potent anticancer drug for these cells. For RKO cell line, the GI_50_ value was measured as 154 μg/ml that was above 100 μg/ml, but at 333.33 and 205 μg/ml concentration the extract was found to approach the LC_100_ and LC_50_. In contrast to this result, the methanol extract never approached the LC_50_ up to concentration level 333.33 μg/ml in HepG2 cells. Hence, it was clear that this extract was acted like an anticancerous agent for both HepG2 and RKO cells under our study.

After confirming the cytotoxic and anticarcinogenic effects of *S. porticalis* methanol extract in HepG2 and RKO cell lines, we further carried out the *in vivo* study to evaluate the biological activity of the algal extract in animal model. Here, *S. porticalis* methanol extract provided protection from the hypoxia-induced oxidative stress and damage to organ systems. It was also found to accelerate the onset of adaptative changes in rats following hypoxic exposure. The bioactive phytochemicals of the extract could have acted synergistically to produce an optimum protective effect under hypoxic stress. The scarcity of oxygen causes impairment of electron transport chain in mitochondria and incomplete reduction of oxygen in hypoxic condition bring about elevated production of free radicals. Previous reports have shown increased level of oxidative stress during exposure to hypobaric hypoxia [106,107] and depleted endogenous antioxidant enzyme level [108,109]. The results of the present investigation also corroborate with the previous reports, where sharp rise in free radical levels and consequent decline of endogenous antioxidant status was seen in animals following exposure to hypobaric hypoxia for 7 days. Administration of *S. porticalis* methanol extract during exposure to hypobaric hypoxia decreased free radical levels and oxidized glutathione (GSSG) concentration, and also increased concentration of endogenous reduced glutathione (GSH) and GSH/GSSG ratio. The antioxidant action of the algal extract could be attributed to the rich content of polyphenols, flavonoids, proanthocyanidins, fatty acid esters, sterols, unsaturated alcohols and other bioactive components. The algal extract induced augmentation of endogenous antioxidant GSH level and GSH/GSSG ratio in blood proves the efficacy of bioactive components in modulating glutathione biosynthesis and stabilization under hypoxic condition. However, the underlying mechanism of such regulatory effects of *S. porticalis* methanol extract on endogenous glutathione biosynthesis needs further investigation at cellular and molecular level.

As discussed previously, a number of studies have been conducted by several investigators to assess the medicinal and therapeutic effects of freshwater algae and diverse group of bioactive compounds were discovered from this vital natural resource. However, there is limited information available regarding the biological and medicinal potential of *S. porticalis* that limits the scope to compare our results with previous reports specific to this alga [110]. To the best of our knowledge, this is the first report on the medicinal potential of *S. porticalis* from the trans-Himalayan Ladakh region that could lead us to develop novel therapeutic compounds from this hitherto untapped unique bioresource.

## Conclusion

The freshwater filamentous alga *Spirogyra porticalis* from the Indian trans-Himalayan cold desert of Ladakh had been identified by morpho-anatomical characteristics and the antioxidant capacities of the algal extracts reported for the first time. The present study revealed high antioxidant capacities and considerable amount of total phenolic compounds in *S. porticalis* extracts which could be used as an efficient natural source of antioxidants. Amongst all tested extracts, the methanol extract was found to possess highest antioxidant capacity and phenolic compounds and this extract was further investigated for GC/MS chemometric profiling, cytotoxic action on human carcinoma cell lines and *in vivo* biological action on animal model. In total, the accession of the study lies within methanol extract of *S. porticalis* which was found to have high antioxidant properties, phenolic content, diverse bioactive phytochemotypes, anticarcinogenic effects along with *in vivo* anti-hypoxic and anti-stress potential. It is evident from the study that the trans-Himalayan alga *S. porticalis* is a reservoir of novel compounds and could be sustainably utilized as an immense wealth for the discovery of novel drugs against a variety of human ailments, specifically oxidative stress-induced disorders.
